# The first feline and new canine cases of *Thelazia callipaeda* (Spirurida: Thelaziidae) infection in Hungary

**DOI:** 10.1186/s13071-018-2925-2

**Published:** 2018-06-08

**Authors:** Róbert Farkas, Nóra Takács, Mónika Gyurkovszky, Noémi Henszelmann, Judit Kisgergely, Gyula Balka, Norbert Solymosi, Andrea Vass

**Affiliations:** 1 Department of Parasitology and Zoology, University of Veterinary Medicine, Budapest, Hungary; 2 Veterinary Hospital, Mohács, Hungary; 3 Irisz Optika Kft, Szolnok, Hungary; 4 Department of Pathology, University of Veterinary Medicine, Budapest, Hungary; 5 Centre for Bioinformatics, University of Veterinary Medicine, Budapest, Hungary; 6 Népkerti Veterinary Clinic, Miskolc, Hungary

**Keywords:** *Thelazia callipaeda*, Dogs, Cats, Vector-borne diseases, Zoonosis

## Abstract

**Background:**

In Europe, the first *Thelazia callipaeda* infections were found in the eyes of some dogs in Italy three decades ago. Since that time, this vector-borne nematode species has been diagnosed in domestic and wild carnivores and humans in some western European countries. During the last few years, autochthonous thelaziosis of dogs, red foxes, cats and humans has also been reported from eastern Europe. The first cases of ocular infections caused by *T. callipaeda* have been described in dogs living in the eastern and southern part of Slovakia and Hungary.

**Methods:**

Whitish parasites found in the conjuctival sac and/or under the third eyelid of one or both eyes of animals were removed and morphologically identified according to species and sex. To confirm the morphological identification with molecular analysis a single step conventional PCR was carried out.

**Results:**

A total of 116 adult worms (1–37 per dog, median: 7, IQR: 14.5 and 7 from a cat) were collected from the eyes of 11 animals. Nematodes were identified as *T. callipaeda* according to the morphological keys and molecular analysis. The sequences of a portion of the mitochondrial cytochrome *c* oxidase subunit 1 (*cox*1) gene were identical to those representing *T. callipaeda* haplotype 1, previously reported in neighbouring and other European countries. Since the infected cat and dogs had never travelled abroad, all of the cases were autochthonous thelaziosis.

**Conclusions:**

The present study reports the first case of thelaziosis in a cat and new cases in 10 dogs found in the southern and northern region of Hungary, respectively. Further studies are needed to clarify whether wild carnivores (e.g. red foxes, golden jackals) may act as reservoirs of this eyeworm species in the country.

## Background

*Thelazia callipaeda* (Spirurida: Thelaziidae) has been known for a long time as the “oriental eyeworm” due to its original geographical distribution in the Far East where it is widely distributed [1]. The adult worms and the first stage larvae parasitize the conjunctival sac of dogs, cats, wild carnivores [foxes (*Vulpes vulpes*), wolves (*Canis lupus*), wild cats (*Felis silvestris*), golden jackals (*Canis aureus*), beech martens (*Martes foina*), racoon dogs (*Nyctereutes procyonoides*) and lynx (*Lynx lynx*)], wild European rabbits (*Oryctolagus cuniculus*), brown hares (*Lepus europaeus*) [2–5] and sometimes humans [6–8]. *Thelazia callipaeda* is responsible for a range of subclinical to clinical signs such as conjunctivitis, lacrimation, ocular discharge or keratitis in animals and humans [9–11]. The lachryphagous *Phortica variegata* (Drosophilidae, Steganinae) has been confirmed as the vector for this spirurid nematode species in Europe [12–15] where the first presence of *Thelazia callipaeda* was found in the Piedmont region of Italy in 1989 [2]. Since that time, an increasing number of imported and/or autochthonous cases of thelaziosis in dogs, red foxes, cats, lagomorphs [2, 3, 5, 16–19] and humans [6–8, 5, 11–20] have been reported in some western European countries. During the last few years, this eyeworm infection of animals and humans has been reported from eastern Europe [7, 8, 21–25]. Recently, the first cases of ocular infections caused by *T. callipaeda* have been described in dogs and red foxes found in the eastern part of Slovakia [26, 27]. *Thelazia callipaeda* was found for the first time in a dog living in the southern part of Hungary [25, 28].

The present paper reports the first autochthonous thelaziosis in a cat and new ocular worm infections of ten dogs caused by *T. callipaeda* found in the northern area of Hungary.

## Methods

### Sample collection

Between October 2015 and December 2017, 11 cases of ocular worm infection in a cat and 10 dogs were signalled by practitioners to the Department of Parasitology and Zoology, University of Veterinary Medicine, Budapest. The cat was a one-year-old male of the European shorthair breed. Affected dog breeds were German Shepherd, Rottweiler, Collie, Hanoverian Hound, Hungarian Vizsla and crossbreeds, aged from 0.8 to 14 years, mostly males except for one (Table 1). Each animal was born in Hungary. All animals examined spent most or all of their time outdoors. The cat and dogs’ owners confirmed that their pets had never travelled abroad.

**Table 1:** Data on the cat and dogs infected with *Thelazia callipaeda*

Locality	Coordinates	Species	Breed	Age (years)	Sex	Sampling date	Affected eye	No. of adult worms female/male (total)
Mohács	45°59'35"N, 18°40'59"E	Cat	European Shorthair	1	Male	October 2015	Right	5/2 (7)
Sajómercse	48°14'46"N, 20°24'53"E	Dog	Collie	5	Male	September 2016	Right	1/0 (1)
Sajógalgóc	48°17'34"N, 20°31'56"E	Dog	Rottweiler	0.8	Male	August 2017	Both	2/0 (2)
Miskolc	48°3'57"N, 20°44'28"E	Dog	German Shepherd	6	Male	August 2017	Left	2/0 (2)
		Dog	Mixed	14	Male	August 2017	Both	1/1 (2)
		Dog	German Shepherd	10	Male	August 2017	Both	7/0 (7)
		Dog	Mixed	8	Male	October 2017	Both	21/16 (37)
		Dog	Hungarian Vizsla	6	Male	December 2017	Both	15/4 (19)
Sajószentpéter	48°13'7"N, 20°42'33"E	Dog	Mixed	1	Male	September 2017	Right	8/8 (16)
Kazincbarcika	48°3'57"N, 20°44'28"E	Dog	Mixed	12	Male	September 2017	Left	9/8 (17)
Budapest	47°30'04"N, 19°03'11"E	Dog	Mixed	7	Castrated female	December 2017	Both	5/1 (6)

### Clinical examination, parasite collection and treatment

On clinical examination after administration of a local anaesthetic, threadlike motile whitish parasites were observed in the conjunctival sac and/or under the third eyelid of one or both eyes of each animal. The worms were removed using sterile cotton swabs or fine forceps and the conjunctival sacs were thoroughly flushed with sterile physiological saline solution. The parasites were collected into 70% ethanol and sent to the Department of Parasitology and Zoology for morphological and molecular identification.

The dogs were treated with a spot-on application of 10% imidacloprid and 2.5% moxidectin (Advocate Spot-On; Bayer HealthCare, Leverkusen, Germany) or Milprazon tablets (milbemicin-oxim 2.5 mg and praziquantel 12.5 mg, KRKA, Novo mesto, Slovenia). The cat did not receive any anti-parasitic treatment.

### Morphological and molecular identification of the worms

Nematodes collected from the eyes of each animal were morphologically identified according to species and sex under a light microscopy combined with a camera (EURmex HD II with IS Capture software) using the previous descriptions [29]. To confirm the morphological identification with molecular analysis a single step conventional PCR was carried out. Genomic DNA was extracted from one or more specimen of worms from each animal, with the NucleoSpin Tissue Kit (Macherey-Nagel GmbH & Co. KG, Düren, Germany) according to the manufacturer’s instructions. An approximately 689 bp partial fragment of the mitochondrial cytochrome *c* oxidase subunit 1 gene (*cox*1) was amplified with the primer pair NTF (5'-TGA TTG GTG GTT TTG GTA A-3') and NTR (5'-ATA AGT ACG AGT ATC AAT ATC-3') [30]. The PCR reaction was carried out in a final volume of 25 μl of mixture containing 0.5 U (0.1 μl) HotStarTaq Plus DNA polymerase (Qiagen, Hilden, Germany), 2.5 μl 10× CoralLoad Reaction buffer (including 15 mM MgCl_2_), 0.5 μl PCR nucleotide Mix (0.2 mM each), 0.5 μl (1.0 μM final concentration) of each primer, 15.9 μl ddH_2_O and 5 μl of template DNA using the following thermal conditions: an initial denaturation step at 95 °C for 5 min followed by 40 cycles of denaturation at 95 °C for 30 s, annealing at 46 °C for 1 min and extension at 72 °C for 1 min. Final extension was performed at 72 °C for 10 min. Reaction mixture without adding template DNA served as a negative control and sequenced *Thelazia callipaeda* DNA (confirmed by sequencing) was used as a positive control to test the efficacy of the reactions. PCR products were electrophoresed in 1.5% agarose gel and visualized under UV-light. Positive amplification products were purified and submitted for Sanger sequencing to the Hungarian Academy of Sciences, Biological Research Centre, Szeged, Hungary. Obtained sequences were manually edited and then aligned and compared to reference GenBank sequences by the nucleotide BLASTN program (https://blast.ncbi.nlm.nih.gov). Representative sequences were submitted to GenBank.

## Results

**Fig. 1:**
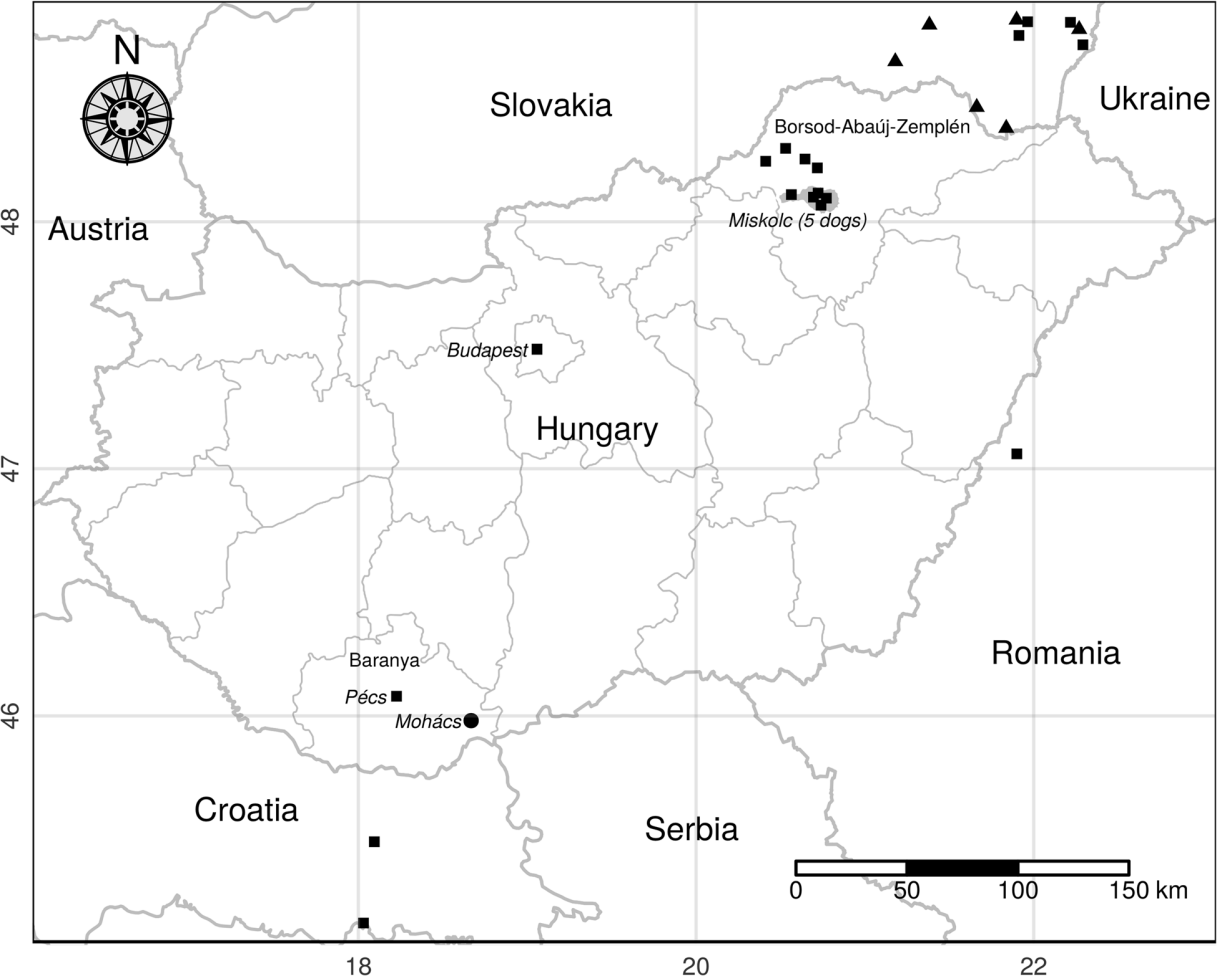
*Thelazia callipaeda* found in a cat (circle), dogs (squares) and red foxes (triangles) in Hungary and close to its borders with neighbouring countries

Nine out of ten infected dogs were kept in five settlements of Borsod-Abaúj-Zemplén county and one animal in Budapest (Fig. 1). Except for two dogs, the others were taken by the owners many times for walking to Bükk National Park located in the north-eastern part of Hungary.

For the cat and all dogs, conjunctival hyperaemia and ocular purulent discharge due to conjunctivitis were observed in one or both eyes. The third eyelid protrusion occurred in one dog. The ophthalmological examination revealed the presence of motile nematodes on the eye surface, in the conjunctival sac and/or under the third eyelids of one or both eyes. A total of 116 adult worms (1–37 per dog, median: 7, IQR: 14.5 and 7 from a cat) were collected from 11 infected animals (Table 1). All specimens recovered were morphologically identified as *T*. *callipaeda* adults. The eyes of four and six dogs were infected with females and parasites of both sexes, respectively (Table 1). Four weeks after removing the worms and starting treatment, the infection had resolved in each animal. Their eyes appeared normal, and no parasites were observed.

The *cox*1 sequences obtained in this study from the cat (GenBank: KX372681.1) and dogs (GenBank: MG677577, MG677578, MG874787, MG874788, MG874790-MG874792, MH178370, MH178371) showed a 100% identity to the sequence of T*. callipaeda* haplotype-1 (GenBank: AM042549.1) reported by Otranto et al. [30] and isolated from dogs in Romania (GenBank: KP087796) and Slovakia (GenBank: KY476400).

## Discussion

In Europe, the first documented *T. callipaeda* infections were found in the eyes of some dogs in Italy three decades ago [2]. In the following decades our knowledge about the distribution of this vector-borne eyeworm species in Europe has greatly expanded. Autochthonous and/or imported thelaziosis affecting domestic and/or wild carnivores has been reported in Germany [16], France [17], Portugal [31–33], Switzerland [18, 34], Spain [19] and Italy [3]. *Thelazia callipaeda* infection has also been found in wild European rabbits [5]. The human ocular thelaziosis reported from Italy and Spain [6, 20] confirms the medical importance of this nematode species in Europe. The occurrence of this spirurid nematode species has also been reported in Eastern Europe. The adult worms have been found in one or both eyes of dogs, cats, foxes and/or humans in Croatia [7, 21], Serbia [8, 22, 35], Romania [23, 24], Bosnia and Herzegovina [21], Bulgaria [25] and Slovakia [26, 27]. Before this study, only a single canine ocular thelaziosis was reported in Hungary [28]. In January 2014, a ten-year-old, male poodle dog with unilateral chronic conjunctivitis and epiphora was presented to a veterinary practice in Pécs, Baranya county, located in the southern part of the country (Fig. 1). The worms were identified as *T. callipaeda* both morphologically and molecularly [25, 28]. However, there has been no explanation of how this dog acquired this eyeworm infection. A year later, in October 2015, seven adult worms were removed from the right conjunctival sac of a one-year-old male cat in Mohács (Fig. 1). This town is 36 km away from Pécs where the infected dog was examined. Ocular worm infections caused by *T. callipaeda* were found in nine dogs originating from five settlements (Table 1 and Fig. 1) of Borsod-Abaúj-Zemplén county. The specimens of this spirurid worm species were found in both eyes of a dog kept in Budapest at the end of 2017. The cat and all the dogs had autochthonous thelaziosis because they had no history of travelling abroad. The infections presented here confirm the parasite’s establishment in two areas of the country. Like in other European countries [12, 19, 32] most Hungarian cases have been diagnosed in late summer and autumn relating to the maximum activity of the vector [15, 36]. The dogs found to be infected with *T. callipaeda* adults in December became infected in summer or earlier because the third stage larvae released by flies in the spring and/or late summer will reach the adult stage many weeks later [9]. It has been experimentally demonstrated that *T. callipaeda* survives at least nine months in definitive hosts [1, 12].

Considering the sporadic cases of feline thelaziosis reported in Europe [17, 21, 22, 32, 35] (except for Switzerland [34]) it is not surprising that only one cat was found in Hungary with a confirmed *T. callipaeda* infection*.* Scientists related the occurrence of rare feline eyeworm infections with the intensive cleaning habits of cats resulting in less contact with the vectors [34]. On the other hand, difficulties in inspecting cats’ eyes by practitioners are also assumed [37]. According to the former dipterological studies, *Phortica variegata*, the vector species of *T. callipaeda* occurs in Hungary [38, 39] where it finds suitable habitat predicted earlier by a geoclimatic model [15]. It is unknown how long *T. callipaeda* has been present in Hungary. This vector-borne nematode species was probably imported to the country in the last decade. It is unlikely that the parasites arrived from abroad with vectors because fruit flies are not good flyers, and they are not known to disperse by wind [40]. Considering the first cases of canine thelaziosis found close to the southern and northern borders of Hungary, we assume that *T. callipaeda* may have been introduced recently or arrived in the country several years ago by infected host(s) from neighbouring countries where the occurrence of this nematode species has been reported [21, 22, 26, 27]. *Thelazia callipaeda* seems more likely to be transmitted to Hungary by wild carnivores, which play an important role in the sylvatic cycle of this eyeworm species [3, 21, 24, 37]. It has been reported that the specimens of this nematode were found in the eyes of red foxes in the northern regions of Balkans [21] and in the southern part of Slovakia close to the Hungarian border [27] (Fig. 1). Another possible explanation is also plausible. *Thelazia callipaeda* might have arrived in Hungary by infected dogs, such as hunting dogs that spent time in a thelaziosis-endemic area. Although there have been some hypotheses of how this eyeworm species could have arrived in the country, the origin and spreading of *T. callipaeda* remains unclear because the haplotype 1 reported in the neighbouring [22–24] and other [19, 21, 31–33] countries has also been found in this study. Our findings also confirm the low genetic variability of this parasite species in Europe [30].

Based on the results of this study, the most urgent need is for improving the knowledge of the local veterinarians who have been largely unaware of this eyeworm infection of dogs and cats. Besides preventative measures, correct diagnosis and appropriate treatment of infected dogs and cats, the veterinarians are also responsible for preventing this parasite infection of pet owners with education. Due to the potential risk of human infestation, physicians and medical ophthalmologists must be informed about the occurrence of this zoonotic parasite in the endemic regions. To date, we have no information about *T. callipaeda* infection of wild canids in Hungary. Therefore, research is needed to clarify whether red foxes and golden jackals act as reservoirs and spreaders of this eyeworm species.

## Conclusions

To the best of our knowledge, this is the first report on the detection of *T. callipaeda* in a cat in Hungary. After reporting the first case of thelaziosis in a dog living in the southern part of the country, ten autochthonous canine thelaziosis have been found in the northern region of Hungary. Further studies are needed to clarify whether wild carnivores (e.g. red foxes, golden jackals) may act as reservoirs of the parasite species in the country.
